# Outcomes of NSTEMI Admissions and Significance of TIMI Scores: A Nationwide Analysis Using the National Inpatient Sample

**DOI:** 10.3390/jcm14010171

**Published:** 2024-12-31

**Authors:** Vivek Joseph Varughese, James Pollock, Prem Patel, Chandler Richardson, Cara Joseph, Dominic Vacca, Hata Mujadzic

**Affiliations:** Prisma Health, University of South Carolina School of Medicine, Columbia, SC 29601, USA; prem.patel5@prismahealth.org (P.P.); chandler.richardson@prismahealth.org (C.R.); cara.joseph2@prismahealth.org (C.J.); dominic.vacca@prismahealth.org (D.V.); hata.mujadzic@prismahealth.org (H.M.)

**Keywords:** NSTEMI, TIMI score, left heart catheterization

## Abstract

**Background/Objectives**: The main aim of this study is to analyze the outcomes of NSTEMI admissions and test the relevance of TIMI as a risk score in a real-world setting. We also examine any potential social or health care disparities involved with outcomes of NSTEMI admissions. This study also investigates factors associated with mortality in NSTEMI admissions and its correlation with heart catheterization during admission. **Methods**: NSTEMI admissions were analyzed using the National Inpatient Sample. TIMI scores were calculated for the admissions and their association with all-cause mortality was studied. Differences in mortality outcomes based on heart catheterization during admission were studied in the subgroup with a TIMI score > 2. Correlations between time to heart catheterization and all-cause mortality in NSTEMI admissions were analyzed. **Results**: No significant social or healthcare disparities were noted among outcomes for NSTEMI admissions. NSTEMI admissions with a calculated TIMI score > 2 had a statistically significant association with all-cause mortality during admission: odds ratio 1.33 (95% CI 1.23–1.43, *p* value: 0.00). The prevalence of mortality among NSTEMI admissions with a calculated TIMI score > 2 who did not undergo heart catheterization was higher with statistical significance (6.23%; 95% CI: 5.84–6.65%), when compared to admissions who underwent heart catheterization (2.41%; 95% CI: 2.25–2.59%). NSTEMI admissions with a calculated TIMI score > 2 who underwent catheterization on or beyond the fourth day of hospitalization had a statistically significant association with all-cause mortality during admission: odds ratio: 2.56 (95% CI: 2.31–2.83, *p* value: 0.00). Age at admission, presence of CKD and CHF were associated with mortality in NSTEMI admissions, with statistical significance. **Conclusions**: Based on the results of our analysis, no disparities in terms of race/sex/hospital location, etc., were seen for NSTEMI in-hospital outcomes. Admissions with a TIMI score > 2 had a statistically significant association with in-hospital mortality, after accounting for confounders. Among NSTEMI admissions with a TIMI score > 2, those who did not undergo heart catheterization during admission were found to have a higher prevalence of in-hospital mortality. No social or healthcare disparities were seen among admissions with TIMI score > 2 who did not undergo heart catheterization during admission. We also noted that NSTEMI admissions with a TIMI score > 2 who underwent heart catheterization on or beyond Day 4 of the initial admission were associated with higher mortality.

## 1. Introduction

Non-ST Segment Elevated Myocardial Infarction (NSTEMI), Unstable Angina (UA) and ST Segment Elevated Myocardial Infarction (STEMI) are the three presentations of acute coronary syndromes (ACS). Advancements in cardiac care units and revascularization techniques have improved patient outcomes in NSTEMI over the years; however, NSTEMI still remains one of the leading causes of death in the United States. In patients presenting with NSTEMI, management can be either invasive (via invasive coronary angiography without prior imaging) or conservative (initial stress testing or anatomic imaging followed by invasive angiography). In general, trials show that an invasive strategy decreases the risk of MI but does not reduce the risk of death when compared to selective angiography. In clinical practice, the decision to follow an early invasive strategy or an ischemia-directed strategy can have a profound impact on patient outcomes. The GRACE and TIMI risk scores are two separate indices with extensive validation data that can guide decisions between an early invasive and an ischemia-directed approach. The TIMI risk score for NSTEMI is a simple prognostication tool that enables a clinician to categorize a patient’s risk of death and ischemic events at the critical initial evaluation. TIMI (thrombolysis in myocardial infarction) scores are calculated with one point each for age > 65, presence of >3 risk factors for coronary artery disease, known coronary artery disease with more than 50% stenosis, two or more episodes of angina in the past 24 h, elevated cardiac markers, ST changes, and aspirin use in the past 7 days. As per the current literature, NSTEMI admissions with a calculated TIMI score > 2 would benefit from invasive strategy, while the decision between early invasive and delayed invasive strategies would depend again on the acuity of clinical features. Patients with ongoing chest pain, development of heart failure and cardiogenic shock would benefit from an early invasive strategy, while for patients with an elevated TIMI score with less acute symptoms, invasive strategies like cardiac catheterization are usually performed before hospital discharge. For patients with TIMI score < 2, ischemia-directed strategies might be favorable, which would involve non-invasive stress testing, either before discharge or in the outpatient setting, as well as invasive strategies like cardiac catheterization, depending on the results of the stress test. In trials that evaluated the routine use of risk stratification scoring, the impact of scores was variable, and rates of death and MI were comparable to usual care. Since patients with ACS are at increased risk of death and nonfatal cardiac events, clinicians must assess prognosis on an individual basis to formulate plans for evaluation and treatment. In real-life practice, this notion becomes more complicated by the multitude of variations in which acute coronary syndromes can present. Although the general description of acute coronary syndrome is the classic left-sided anginal chest pain with radiation, worsened by activity and relieved with rest, more than often, there are presentations that vary from this. Anginal equivalents, epigastric and shoulder pain, dizziness, etc., are examples for the same. Interprovider variability in assessment of the symptoms can be a hindrance towards the standardized management of the condition. It is true that clinical decision making is the cornerstone of medical practice, but large-scale population-based studies based on objective scoring scales and related outcomes can be very helpful for the providers in shaping their clinical decision making. In a retrospective review by Garcia et al. [1], it was found that two-vessel disease was more likely found in patients with TIMI scores of 3–4 than in patients with TIMI scores of 0–2. Additionally, triple-vessel or left main disease was more likely found in patients with TIMI scores of 3–4 than in patients with TIMI scores of 0–2. Looking at the data from various clinical trials and meta-analyses, the timing of the revascularization strategy plays a critical role in determining outcomes of ACS admissions. IMACS was one of the largest clinical trials analyzing the outcomes of early versus delayed angiography and outcomes in NSTEMI patients [2]. The outcomes of the study proved that an early intervention strategy (median 14 h since admission) was not superior to a delayed intervention strategy (median 50 h since admission) for prevention of in-hospital death, myocardial infarction or stroke, but early intervention had superior outcomes in preventing long-term mortality and ischemic events.

The decision between an early and a delayed invasive strategy is purely based on the clinical expertise of the provider, but having a standardized protocol to select patients who would benefit from an ischemia-directed approach compared to a delayed invasive approach can be beneficial in the long-term management of NSTEMI admissions. While the decision concerning an invasive versus an ischemia-directed approach may not produce differences in terms of long-term mortality, it surely has a considerable effect on reducing subsequent myocardial infarction and hospital mortality. A delayed invasive approach compared to an ischemia-directed approach is a decision made after the acute presentation subsides and tends to be more applicable to the discharge planning for the patient. A standardized protocol at this point, with a proven objective score to make the decision for either a delayed invasive or an ischemia-directed approach, could have favorable outcomes on subsequent MIs and related deaths. The main aim of our study is to create a large patient pool by selecting admissions for NSTEMI across the United States, and analyzing how the invasive approach (whether early or delayed) based on the TIMI risk score translates into hospital mortality outcomes.

## 2. Materials and Methods

The aim of this study was to analyze outcomes of NSTEMI admissions across the United States among those aged 18 years or older using the National Inpatient Sample (NIS) 2019. STATA 18 was used to extract and analyze the data. ICD 10 codes were used for identifying patients admitted with NSTEMI (ICD 10 code base code 1214) [3], and the ICD 10 PCS (y840) [3] code was used to identify NSTEMI admissions who underwent heart catheterization during admission.

The primary population study consisted of crude analysis of NSTEMI admissions and associated mortality. The total admission pool was divided based on the in-hospital mortality outcomes of the admissions. The objective was to look for any significant social or healthcare disparities among the survivors versus non survivors of NSTEMI. The population characteristics of NSTEMI admissions were studied, and the characteristics of patients who died during hospitalization were compared to the whole population of NSTEMI admissions.

For subsequent analysis, we calculated the TIMI scores for each of the admissions. Age, presence of CAD (>50% stenosis), long-standing aspirin use, >3 risk factors for CAD, and troponin elevation were used to calculate TIMI score for each NSTEMI admission. Age > 65, presence of CAD (ICD 10 base code 1251), documented aspirin use (ICD 10 cod Z7982), >3 documented risk factors (hypertension, diabetes hypercholesterolemia, smoking: elixir codes were used) and troponin elevation (ICD 10 code R7989) were given a point each to calculate the TIMI score for each of the admissions. EKG changes and anginal episodes in the past 24 h were parts of the TIMI score not used in the calculation. This is based on the assumption that both these factors can be subjective, and their exclusion will only understate the implication of the TIMI score. Moreover, the nature of ICD codes for each of these diagnoses would create bias based on the nature of the NIS database.

From the total pool of NSTEMI admissions, patients who had a calculated TIMI score > 2 and did not undergo catheterization during the specific hospital admission were identified as a subgroup (based on the PCT 10 code y840 for heart catheterization). NSTEMI patients with a TIMI score ≥ 2 and who died during the admission were excluded as absence of catheterization could represent bias due to early mortality.

The next part of our study involved analyzing the association between a calculated TIMI score > 2 and mortality for the NSTEMI admissions. We used probit logistic regression for the analysis. Mortality was defined as the dependent variable. The confounders included in the multivariate regression included age, previous myocardial infarction (ICD 10 code I1252), heart failure (elixir code for CHF), arrhythmias (elixir code for arrhythmias), race, region of admission and monthly income, which were the other independent variables along with the TIMI score used in the regression. A two-tailed *p* value < 0.05 was used to determine statistical significance.

We sub-grouped NSTEMI admissions with a TIMI score > 2 based on documented heart catheterization during admission. The proportion of all-cause mortality was analyzed in both the groups to look for statistically significant differences. The population characteristics of the subgroup with a TIMI score > 2 that did not undergo catheterization were investigated based on gender, race, region, monthly income, hospital bed size, and primary expected co-payer to identify any social or healthcare disparities.

In the subgroup of NSTEMI admissions with calculated TIMI scores > 2, we studied the association between the day of revascularization (assuming admission day as day 0) and mortality during admission. Multivariate probit logistic regression was used for the analysis. Mortality was the dependent variable defined in the NSTEMI group with a TIMI score > 2. Admissions who underwent catheterization on Day 1, Day 2, Day 3 and Day 4 or beyond were separately analyzed. Age, previous myocardial infarction (ICD 10 code I1252), heart failure (elixir code for CHF), arrhythmias (elixir code for arrhythmias), race, region of admission and monthly income were the other independent variable scores used in the regression. A two-tailed *p* value < 0.05 was used to determine statistical significance.

Among the NSTEMI admissions with a calculated TIMI score > 2, the association between comorbid health conditions and mortality was analyzed using the chi-square test. *p* value < 0.05 was used to determine statistical significance.

## 3. Results

**Figure 1:**
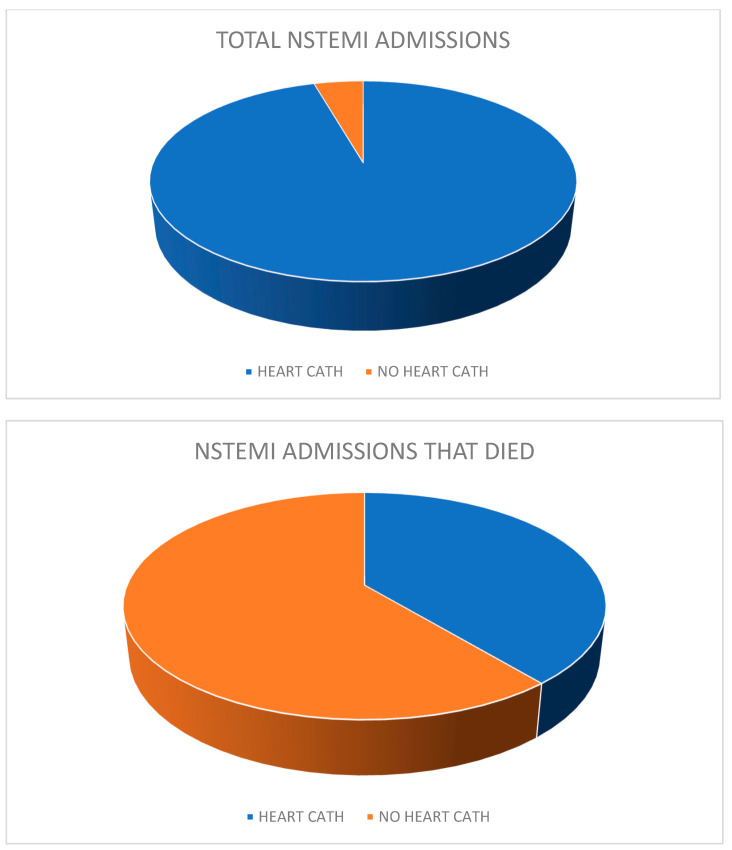
NSTEMI admissions and heart catheterization.

Using the NIS, 459,360 admissions for NSTEMI were identified. Of these admissions, 14,485 patients died during the hospitalization. Analyzing the population characteristics of NSTEMI admissions and comparing them with those of NSTEMI admissions who died during the hospital stay (Table 1), no social or healthcare disparities were found. The median household income of the patient, race, sex, primary expected co-payer, region, bed size, location, and ownership of the admitting hospitals was the same across both the groups. The percentage of admissions that occurred over a weekend was also the same across both groups. The mean age of NSTEMI admissions among patients who died was greater (mean age 75.57 years (75.15–75.99)) than the mean age of all NSTEMI admissions generally (mean age 67.81 years (67.69–67.93)). It was also noted that only 5645 admissions (38.97%) of patients who died during the NSTEMI hospitalization had a heart catheterization conducted sometime during their inpatient stay, compared to 315,672 admissions (68.72%) among the total general NSTEMI admissions pool (Figure 1).

Out of the total NSTEMI admissions, we identified 64,044 admissions with a calculated TIMI score > 2. Among these admissions, 16,582 (25.89%) died during the hospitalization. This was higher compared to the 14,485 deaths (3.15%) in the general NSTEMI admission population. In fact, a calculated TIMI score > 2 on presentation showed a statistically significant association with mortality during the same admission, with an odds ratio of 1.33 (95% CI: 1.23–1.43) (Table 2).

**Figure 2:**
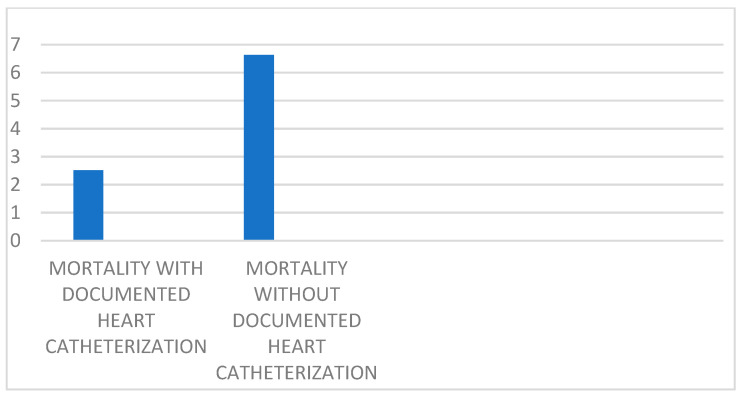
Mortality proportions in NSTEMI admissions with TIMI > 2.

Among the NSTEMI admissions with a TIMI score > 2, those with and without the documented procedure of left heart catheterization during admission were subdivided, and the proportion of mortality during admission was analyzed in both groups. Amongst the NSTEMI admissions with a calculated TIMI score > 2, the subpopulation that underwent heart catheterization during admission had a proportion of mortality at 2.518 (2.3502–2.5982), while the prevalence of mortality in the subpopulation of admissions with a calculated TIMI score > 2 that did not undergo catheterization was 6.636 (5.849–6.754). The prevalence of mortality in the subgroup that did not undergo catheterization during admission was higher with statistical significance than the subgroup that did undergo catheterization during admission (Table 3, Figure 2).

The subpopulation of NSTEMI admissions with a calculated TIMI score > 2 who did not undergo catheterization during admission was sub-grouped and analyzed for social and healthcare disparities. (Patients who died during admission were excluded as mortality could be a confounding factor for not undergoing heart catheterization.) No significant disparities in social or healthcare factors were found in this group compared to the total NSTEMI admission population (Table 4). The primary expected co-payer was Medicare in 53,156 (83%) of the admissions in this population compared to Medicare being the primary co-payer in 277,085 (60%) of the total NSTEMI admissions, but the mean age of the subgroup was also higher compared to the total NSTEMI admissions.

The association between mortality during admission for NSTEMI admissions (with TIMI score > 2) and documented time to catheterization (documented in days, considering the admission day as “Day 0”) was also analyzed using liner regression analysis. The analysis was performed after accounting for age, sex, race, quarterly income, and coexisting diagnosis of congestive heart failure (CHF). The NSTEMI admissions were further grouped into admissions who received heart catheterization on Day 1, Day 2, or Day 3 and Day ≥ 4 of admission, and the association of each of the groups with mortality during admission was analyzed. Time to catheterization had a positive correlation with mortality during admission, with a positive coefficient of regression of 0.002 (standard error 0.0003155, *p* value 0.000, 95% CI: 0.001402–0.0026411) in NSTEMI admissions with a calculated TIMI score > 2. Examining the association of in-hospital mortality during admission and the day on which heart catheterization was performed, admissions who had catheterization performed on Day ≥ 4 after admission had an odds ratio of 2.56 (2.31–2.83) (Table 5). NSTEMI admissions who underwent heart catheterization on Day 1, Day 2, or Day 3 did not have any significant association with mortality during admission.

From the general NSTEMI admission population, documented diagnoses that were associated with mortality during hospitalization were analyzed. Age documented at admission, documented diagnosis of CHF, and documented diagnosis of chronic kidney disease (CKD) had significant associations with in-hospital mortality during NSTEMI admission (Table 6). Documented diagnosis of diabetes, smoking, alcohol abuse, or substance abuse did not have any significant association with in-hospital mortality among the general NSTEMI admissions.

## 4. Discussion

When examining the social and healthcare aspects associated with NSTEMI admissions (Table 1), no significant disparity was observed. The only significant finding was an increase in crude mortality for NSTEMI admissions who did not undergo heart catheterization when compared to admissions who did. This result is comparable to findings observed when analyzing similar data from the Canadian ACS Registry II [4]. While analyzing the population characteristics of NSTEMI admissions with a TIMI score ≥ 2 who did not undergo heart catheterization, no notable discrepancies were seen compared to the general NSTEMI admission characteristics. The primary expected co-payer was Medicare in 53,156 (83%) of the admissions in this population compared to Medicare being the primary co-payer in 277,085 (60%) of the total NSTEMI admissions, but the mean age of the subgroup was also found to be higher compared to the total NSTEMI admissions (76.28 as opposed to 67.81). Documented age at diagnosis, presence of co-existing diagnosis of congestive heart failure and CKD were associated with all-cause mortality in NSTEMI admissions, with statistical significance.

The heterogeneity of presentations among patients admitted for NSTEMI confers a wide range of risk for death and ischemic events [5,6,7]. These patients who lack indications for immediate intervention need to have early risk stratification performed, especially in light of the results of seen in the PRECLUDE-2 study, which suggested that even in NSTEMI patients who possess multiple ischemic risk factors, suboptimal management is often delivered [8]. This observation had been previously dubbed the “treatment risk paradox” [9,10,11,12]. To make matters worse, this harsh reality is compounded by the fact that patients with NSTEMI also tend to erroneously receive less aggressive secondary preventative measures compared to their STEMI counterparts [13,14]. The TIMI risk score is a simple prognostic method of stratifying this risk and helps in therapeutic decision making. The Antman et al. study in 2000 first proposed the TIMI score based on seven different independent variables, and the score predicts the outcomes of patients with unstable angina or NSTEMI. A higher TIMI score is associated with an increased number of adverse events at 14 days and has also been correlated with increasingly more severe angiographic coronary disease. In the TACTICS-TIMI 18 study, the outcomes were compared between patients who presented with UA or NSTEMI who underwent either early invasive treatment or conservative medical management. The trial found that patients with moderate to high risk (TIMI score > 2) who underwent early invasive treatment showed a significant decrease in mortality and cardiac complications compared to those who were conservatively managed. Interestingly, there was no significant difference in outcomes in patients with low risk between the invasive and conservative management.

Our study was conducted to assess the outcomes of NSTEMI admissions using the TIMI score in the United States. It also evaluates the association of mortality in moderate- to high-risk NSTEMI admissions (TIMI score ≥ 2) in patients who did and did not undergo heart catheterization during the same hospitalization. Within the subgroup that received invasive intervention, those who were treated earlier (less than 4 days) had better outcomes. The superior prognosis with early revascularization is congruent with prior studies such as the RIDDLE-NSTEMI [15] and ISAR-COOL [16] trials. The TACTICS TIMI 18 trial [17] found that in UA and NSTEMI, an early invasive strategy with the concurrent use of Tirofiban was associated with better outcomes. The FRISC II trial [18] had similar findings which were supportive of early intervention strategies in UA/ NSTEMI. The unfavorable higher mortality in the delayed intervention groups was attributed to prolonged ischemic time leading to irreversible myocardial damage and subsequent necrosis. A 2022 prospective study in the United States included 113 patients who were admitted for a STEMI and found that lower total ischemic times were favorable with regards to mortality. Although there were several limitations to this study compared to our analysis, the findings strongly support that shorter total ischemic times (TIT) can be a good predictor of clinical outcomes. There are also minimal data in patients admitted for an NSTEMI on TIT; therefore, many considerations are extrapolated from data from trials investigating STEMI patients. The use of TIT has growing evidence to support the use of this factor as a quality indicator in combination with the well-established door-to-balloon time.

As mentioned in the Section 1, the decision between a delayed invasive and an ischemia-directed approach becomes crucial in discharge planning for patients admitted with NSTEMI. Based on the results of our study, a calculated TIMI score > 2 has a higher association with in-hospital mortality. This proportion of mortality was significantly higher for patients with a calculated TIMI score > 2 who could not undergo left heart catheterization. This becomes important in making the decision whether to pursue an invasive approach prior to discharge for patients with a calculated TIMI score > 2, rather than an ischemia-directed approach in the outpatient setting. More real-life prospective as well as retrospective studies need to be performed to further stratify the significance of the score and identify caveats that could be relevant to its interpretation.

## 5. Limitations of This Study

NIS database use has been rising due to the ease of access to many subjects and the generalizability of the data. However, there are several limitations when it comes to the use of this type of sample. One main limitation to consider is the lack of surrounding clinical context regarding confounding variables. It becomes more difficult to draw causal inferences from retrospective data due to drug information and the inability to determine variables outside of the hospital or clinic. We have reported the demographics of the patients included in our study to provide objective data when comparing the social disparities. For instance, the reason for a delay in heart catheterization for the patients admitted with an NSTEMI was not reported, whether it was acute renal injury, uncontrolled hypertension, etc. Another limitation of using NIS is the possibility of misclassification of information. The national data is based on claim codes that are easily susceptible to discrepancies from the actual diagnoses, due to, for example, recording codes for payment rather than the true clinical diagnosis. Specifically, in our study, one limitation relates to the criteria of the TIMI risk score. The nature of the NIS database prohibited the inclusion of ST wave changes on the electrocardiogram and severe anginal symptoms in the TIMI risk score calculation. If these factors were included, more patients would fit the moderate-risk (TIMI ≥ 2) group; hence, our study may be under reporting the number of patients that would be included. However, those patients with either or both findings typically have other co-morbidities and would have had a calculated TIMI score ≥ 2, nonetheless. Despite these limitations, further randomized controlled trials on this topic are warranted.

The data representation in this system is based on ICD codes for diagnosis entered by the admitting physician. NSTEMI being a common diagnosis, physician-to-physician variability in terms of the preliminary diagnosis would not be a limiting factor. However, accurate documentation of associated clinical conditions may be subject to physician-to-physician variability.

Another limitation of this study was in determining the calculated TIMI score: the presence of two anginal episodes and EKG changes were two factors not documented in the NIS database. However, since our study focuses on the relevance of a TIMI score ≥ 2 in the management of NSTEMI/UA, exclusion of these two criteria can only underestimate the significance of the score. If we had data for EKG changes and angina documented for the NSTEMI admissions, the cohort of NSTEMI admissions with TIMI scores ≥ 2 would have been higher, and the associations we found for mortality/adverse events would have attained more statistical significance.

This article was previously posted to the med aRxiv preprint server [19] on 27 April 2020.

## 6. Conclusions

The findings of our study strongly suggest that patients with moderate to high-risk NSTEMI admissions (TIMI score > 2) who undergo heart catheterization have better outcomes compared to those who do not. Furthermore, among the patients who undergo heart catheterization, those who receive early revascularization have favorable mortality rates compared to those who have a delay in treatment. Thus, attempts to decrease the TIT should be made to reduce overall mortality rates in patients admitted for NSTEMI. Our analysis did not reveal any social or healthcare disparities among NSTEMI admissions who died during their hospital stay compared to the general NSTEMI admissions. No social or healthcare disparities were found between NSTEMI patients with a TIMI score > 2 who did not undergo heart catheterization during admission and patients with a TIMI score > 2 who underwent heart catheterization during admission.

## Figures and Tables

**Table 1:** Baseline/population characteristics of NSTEMI admissions.

Baseline Characteristics	Total NSTEMI Admissions	NSTEMI Admissions Who Died During the Hospitalization
Mean Age	67.811 (67.69–67.931)	75.57 (75.153–75.994)
Sex	Male: 60.69%	Male: 58.78%
	Female: 39.31%	Female: 41.22%
Race	White: 76.23%	White: 74.23%
	Black: 12.22%	Black: 10.37%
	Hispanic: 8.87%	Hispanic: 8.33%
Median Household Income (calculated using the ZIPCODE region)	1st Quartile: 31.78%	1st Quartile: 31.93%
	2nd Quartile: 26.66%	2nd Quartile: 27.01%
	3rd Quartile: 23.82%	3rd Quartile: 23.25%
	4th Quartile: 17.74%	4th Quartile: 17.81%
Admission over a Weekend	25.86%	27.31%
Hospital Ownership	1. Government, nonfederal: 8.62%	1. Government, nonfederal: 8.8%
	2. Private, not for profit: 75.32%	2. Private, not for profit: 74.42%
	3. Private, investor owned: 16.06%	3. Private, investor owned: 16.78%
Hospital Bed Size	Small: 20.43%	Small: 18.57%
	Medium: 30.51%	Medium: 30%
	Large: 49.06%	Large: 51.43%
Hospital Region	Northeast: 17.16%	Northeast: 17.43%
	Midwest: 22.36%	Midwest: 20.75%
	South: 41.69%	South: 40.91%
	West: 18.8%	West: 20.92%
Primary Expected Co-payer	Medicare: 60.32%	Medicare: 61.56%
	Medicaid: 9.06%	Medicaid: 11.23%
	Private: 23.31%	Private: 21.21%
	Self-pay: 4.09%	Self-pay: 4.51%
Proportion Who Underwent Catheterization	68.72% (68.42–69.01%)	38.97315% (37.2–40.76%)
Total	459,360	14,485

NSTEMI: Non-ST Segment Elevated Myocardial Infarction.

**Table 2:** Association between TIMI score > 2 and all-cause mortality among NSTEMI admissions.

Total NSTEMI Admissions	Association with Mortality During Admission (Odds Ratio)
Calculated TIMI Score > 2	1.43 (1.32–1.52) *p* value = 0.000

NSTEMI: Non-ST Segment Elevated Myocardial Infarction, TIMI score: Thrombolysis in Myocardial Infarction Score: Total NSTEMI admissions were selected, from which the subpopulation with a calculated TIMI score > 2 was selected. (From the admission data, age > 65, presence of more than three risk factors for CAD, presence of CAD, long term use of aspirin and troponin elevation were used in the calculation).

**Table 3:** All-cause mortality proportion among NSTEMI admissions based on the results of left heart catheterization during the hospital stay.

NSTEMI Admissions with TIMI Score > 2	Proportion Died
Subpopulation that underwent catheterization during the current admission	2.518 (2.3502–2.5982)
Subpopulation that did not undergo catheterization during the current admission	6.636 (5.849–6.754)

NSTEMI: Non-ST Segment Elevated Myocardial Infarction, TIMI Score: Thrombolysis in Myocardial Infarction Score.

**Table 4:** Population characteristics of NSTEMI admissions with a TIMI score > 2 who did not undergo left heart catheterization during the hospital stay.

NSTEMI (TIMI > 2) and Did Not Undergo Left Heart Catheterization During Admission	Population Characteristics
Mean Age	76.2861 (76.05096–76.5142)
Sex	Male: 57.97%
	Female: 42.03%
Race	Whites: 73.58%
	Blacks: 11.55%
	Hispanics: 8.4%
Median Household Income (calculated using the ZIPCODE region)	1st quartile: 31.07%
	2nd quartile: 26.52%
	3rd quartile: 24.04%
	4th quartile: 18.36%
Admission over a Weekend	25.39%
Hospital Ownership	Government, nonfederal: 8.51%
	Private, not for profit: 76.23%
	Private, investor owned: 15.26%
Hospital Bed Size	Small: 22.59%
	Medium: 31.26%
	Large: 46.15%
Hospital Region	Northeast: 18.16%
	Midwest: 21.71%
	South: 39.66%
	West: 20.47%
Primary Expected Co-payer	Medicare: 83%
	Medicaid: 3.86%
	Private Insurance: 9.35%
	Self-Pay: 1.52%
Location/Teaching Status of the Hospital	Rural: 9.78%
	Urban non-teaching: 20.52%
	Urban Teaching: 69.7%
All Patient Refined DRG: Severity of Illness Subclass	Minor loss of function: 10.1%
	Moderate Loss of function: 42.86%
	Major loss of function: 30.34%
	Extreme Loss of function: 16.7%
Total	64,044 (61,601.55–66,488.43)

NSTEMI: Non-ST Segment Elevated Myocardial Infarction, TIMI score: Thrombolysis in Myocardial Infarction Score.

**Table 5:** Correlation between time to left heart catheterization and all-cause mortality among NSTEMI admissions with TIMI score > 2.

Day of Admission When Heart Catheterization Was Performed (NSTEMI Admissions with TIMI > 2)	Association with Mortality During Admission (Odds Ratio)
Day 1	0.54325
Day 2	0.50108
Day 3	0.44958
Day 4 and beyond	2.673 (2.314–2.839) *p* value = 0.00; SE: 0.1338

NSTEMI: Non-ST Segment Elevated Myocardial Infarction, TIMI score: Thrombolysis in Myocardial Infarction Score.

**Table 6:** Association between comorbid conditions and all-cause mortality among NSTEMI admissions with TIMI score > 2.

Documented Diagnosis in the NSTEMI Admission Cohort	Association with All-Cause Mortality During Admission: Odds Ratio	*p*-Value	Confidence Interval (95%)
Documented Age	1.062	0.000	1.050–1.057
Congestive Heart Failure	3.534	0.000	3.254–3.841
CKD	2.470	0.000	2.293–2.661
Diabetes	0.650759	0.000	0.591339–0.72789
Alcohol abuse	0.995608	0.974	0.80693–1.23601
Drug abuse	0.48244	0.000	0.35357–0.6582
Smoking	0.450816	0.000	0.39584–0.51352

NSTEMI: Non-ST Segment Elevated Myocardial Infarction, TIMI score: Thrombolysis in Myocardial Infarction Score, CKD: chronic kidney disease.

## Data Availability

The data presented in this study are openly available in [NIS], reference number [2019].
